# Environmental triggers of COPD symptoms: a case cross-over study

**DOI:** 10.1136/bmjresp-2017-000179

**Published:** 2017-07-03

**Authors:** Susan R Sama, David Kriebel, Rebecca J Gore, Rebecca DeVries, Richard Rosiello

**Affiliations:** 1 Department of Public Health, University of Massachusetts Lowell, Lowell, USA; 2 Research Department, Reliant Medical Group, Worcester, USA

**Keywords:** COPD exacerbations, COPD epidemiology, Respiratory infection, Asthma epidemiology

## Abstract

**Introduction:**

This study investigated the hypothesis that common environmental chemical exposures with known irritant or sensitising properties trigger exacerbations for patients with chronic obstructive pulmonary disease (COPD).

**Methods:**

We conducted a case cross-over study in 168 patients with COPD who were members of a disease management group in central Massachusetts. Participants completed a baseline health survey and several short exposure surveys. Exposure surveys were administered by a nurse when a participant telephoned to report an exacerbation (case periods) and at a maximum of three randomly identified control periods when they were not experiencing an exacerbation. We compared exposures in the week preceding an exacerbation with exposures in normal (non-exacerbation) weeks. The questionnaire assessed short-term (1 week) home, community and workplace activities and exposures that may be associated with COPD exacerbation.

**Results:**

Self-reported exercise was negatively associated with exacerbation (OR=0.59, 95% CI: 0.35 to 1.00). Among the environmental chemical exposures, car and truck exhaust (OR=4.36, 95% CI: 1.76 to 10.80) and use of scented laundry products (OR=2.69, 95% CI: 1.31 to 5.52) showed strong positive effects. Self-reported respiratory infections were strongly associated with exacerbation (OR=7.90, 95% CI 4.29 to 14.50). Variations in outdoor temperature were associated with COPD exacerbation risk (moderate versus cold temperature OR=1.95, 95% CI 1.09 to 3.49 and warm versus cold OR=0.43, 95% CI: 0.26 to 0.70).

**Conclusions:**

These results suggest that some environmental chemical exposures may play a role in triggering COPD exacerbations. If confirmed, they may provide useful guidance for patients with COPD to better manage their disease.

**Key messages**

- The key question is whether common environmental chemical exposures with known irritant or sensitising properties trigger exacerbations for patients with chronic obstructive pulmonary disease (COPD).
- Among the environmental chemical exposures we evaluated, car and truck exhaust (OR=4.36, 95% CI: 1.76 to 10.80) and use of scented laundry products (OR=2.69, 95% CI: 1.31 to 5.52) appeared to be associated with increased risk of COPD exacerbation.
- Very few studies have tried to quantify the risk of COPD exacerbation from acute environmental chemical exposures. If these findings are confirmed, they may present an important opportunity for improving the quality of life for patients with COPD.

## Background

Chronic obstructive pulmonary disease (COPD) is the third leading cause of death in the USA, affecting over 10 million people and costing over US$15 billion in healthcare costs each year.^1^ Tobacco smoking is the primary cause of COPD,^2 3^ although occupational exposures to a number of hazardous chemicals and air pollution are also important preventable causes.^4–6^ The most important strategy for disease management is preventing the occurrence of acute episodes of COPD exacerbation.^7 8^ Failing that, rapid medical response at the start of an exacerbation can blunt the damage caused by the cascade of inflammatory processes that are triggered.^9^ Uncontrolled exacerbations are strongly associated with disease progression^10^ and mortality. GOLD (Global Initiative for Chronic Obstructive Lung Disease) defines an exacerbation as ‘an acute worsening of respiratory symptoms that result in additional therapy’.^11^

Despite the well-recognised importance of preventing COPD exacerbation, research on preventable causes of exacerbation has been limited to a relatively small set of factors. The most important trigger of exacerbation appears to be respiratory infection.^12^ There is good evidence that urban air pollution and temperature can increase risk of COPD exacerbation,^13–16^ as can environmental tobacco smoke.^17 18^ Beyond these four factors, little is known about other preventable triggering exposures.

We hypothesised that there may be environmental chemical exposures other than urban air pollution and second-hand tobacco smoke which increase the risk of COPD exacerbation. It is likely that the mechanism by which urban air pollution triggers exacerbations involves inflammatory processes which also underlie other health endpoints including asthma, bronchial hyper-reactivity, acute loss of pulmonary function and cardiovascular disease. Under this assumption, it seems reasonable that other chemical exposures which act through respiratory inflammation and irritation pathways might also trigger COPD exacerbations. These might include volatile organic compounds and fine particulates in indoor air. There are a large number of such chemicals identified as triggers for asthma attacks,^19 20^ and we hypothesised that asthmagens (and particularly irritant asthmagens) may also act as triggers of COPD exacerbations in susceptible patients.

The objective of this study was to investigate a wide range of environmental chemical exposures known to cause respiratory irritation and/or asthma, with the goal of identifying exposures that may trigger COPD exacerbation.

## Methods

All study materials and protocols were approved by the Reliant Medical Group Institutional Review Board.

### Study population

The study population was drawn from a large medical group practice in central Massachusetts. The area centres on Worcester County (population 818 963), comprising 62 towns and the city of Worcester, the third largest city in New England with a population of 181 045.^21 22^ As a part of its efforts to improve patient quality of life, increase compliance with care guidelines and control costs, the group medical practice maintains and staffs a COPD disease management group (DMG). Patients are enrolled into the group by pulmonologists or primary care physicians after a diagnosis of COPD based on symptoms and spirometry.

The purpose of the DMG is to educate patients and teach them to properly manage their disease with the overall goal of reducing the frequency and severity of their exacerbations, thus slowing their disease progression. These patients are followed closely through periodic clinic visits and regular telephone contacts with a nurse manager. Patients come in for clinic visits approximately twice a year. In addition, routine calls are made by DMG nurses to all patients at least once every 4–6 months; more frequent calls and visits are made if patients have unstable COPD. Patients are asked to call their DMG nurse as soon as they notice that they are experiencing an exacerbation so that the DMG nurse can ensure proper treatment. Patients are instructed to increase use of their rescue inhalers when COPD symptoms increase, and if that fails and symptoms (cough, shortness of breath, sputum production) continue to escalate, exacerbations are treated with oral antibiotics and/or steroids. The majority of patients enrolled in DMG have prefilled medications (steroids and antibiotics) kept at home so that they can begin treatment as soon as an exacerbation begins.

We invited participation from patients who were DMG members. All had received a physician’s diagnosis of COPD, had received at least one antibiotic prescription in the 3 years prior to study enrolment, had experienced at least one exacerbation in the 15-month study period and had no other lung diseases (eg, interstitial lung disease, lung cancer, diffuse bronchiectasis) except asthma which is often misdiagnosed in patients with COPD.^23^ We conducted focus groups and recruited patients enrolled in DMG into our case cross-over study. The initial recruitment included informed consent and a baseline health survey followed by exposure surveys during case and control periods which are defined in more detail below. Participants were compensated US$15 for the long baseline health questionnaire and US$10 each for the short exposure questionnaires.

### Focus groups

We conducted two focus groups, each with eight patients recruited from the COPD DMG. The first was used to review our *a priori* list of potential triggers and to ask about others that should be added. The second was used to pilot the exposure survey for readability and formatting. The final exposure survey instrument contained questions on activities and exposures that one or more focus group participants reported could sometimes aggravate COPD symptoms.

### Baseline survey

At the initial telephone recruitment, a standard respiratory questionnaire (modified American Thoracic Society questionnaire)^24^ was administered to gather lifetime information on disease onset, comorbidities, smoking and occupational history.

### Case cross-over exposure survey

The data were collected in a prospective case cross-over study designed to evaluate the relationship between activities and exposure to certain products and activities and exacerbation of existing COPD.^25^ The case cross-over design allows each case to serve as his or her own control. Each patient had at least one case period (COPD exacerbation, defined below) and up to three randomly identified control periods (non-exacerbation weeks). Data were collected over a 15-month period from January 2011 to March 2012. The principal data gathering instrument was a short environmental exposure and symptom questionnaire administered by a nurse when participants telephoned to report an exacerbation and at up to three randomly identified control periods initiated by the study nurse when the patient was not experiencing an exacerbation. This questionnaire assessed short-term (1 week) home, community and workplace activities and exposures that we hypothesised might be associated with COPD exacerbation.^6 19 26–30^

The questionnaire was organised into four main sections: (1) physical activities that might result in chemical exposures (such as raking leaves or cooking with a gas stove); (2) home and community chemical exposures (based largely on standard environmental exposure questionnaires)^31 32^; (3) workplace exposures (based on the American Thoracic Society and European Respiratory Society questionnaire items on vapours, dust, gas and fumes); and (4) non-chemical triggers of COPD (such as upper respiratory tract infections, exercise and weather).

### Exacerbation

Following the treatment plan, patients telephoned the DMG nurse to report that they were starting to use the prefilled medications because of a worsening of symptoms and the nurse clinically confirmed that the patient was indeed experiencing an exacerbation and needed the additional medication. Worsening symptoms were defined following Seemungal^33^ as at least two of the following: increased dyspnoea, sputum volume or purulence AND one symptom of an upper respiratory tract infection (fever, cough or wheeze, tachycardia or tachypnoea). COPD exacerbation was defined as occurring when (1) a patient reported that he/she had begun to take prefilled medications (antibiotics and/or oral steroids) because of a worsening of COPD symptoms that did not resolve with increased use of rescue inhalers and (2) the DMG nurse confirmed that the patient’s symptoms met Seemungal’s definition of exacerbation and therefore required this additional treatment. To ensure that patients were in a steady state during control periods, we required a minimum of 4 weeks between exacerbation periods.

COPD severity was characterised using the GOLD grades.^11^ The GOLD grades are as follows: grade I (mild) FEV_1_/FVC <0.70 and FEV_1_ ≥80% of normal; grade II (moderate) FEV_1_/FVC <0.70 and FEV_1_ 50%–79% of normal; grade III (severe) FEV_1_/FVC <0.70 and FEV_1_ 30%–49% of normal; and grade IV (very severe) FEV_1_/FVC <0.70 and FEV_1_ <30% normal, or <50% normal with chronic respiratory failure present. The majority of these patients had COPD signs and symptoms meeting GOLD grades I– IV. Our prestudy medical chart review found that there were a few DMG participants (7%) with symptoms of COPD but normal FEV_1_/FVC ratios; we did not exclude these from the study because they met our *a priori* case definition of a physician’s diagnosis of COPD (see Study Population).

### Statistical analyses

Statistical analyses were conducted in SAS V.9.3 (SAS Institute). Conditional logistic regression models were used to investigate determinants of the risk of COPD exacerbation (conditional on individual subject IDs), starting with single predictor models. We then used a forward stepwise approach, manually adding variables to multiple predictor models based on goodness of fit (−2log likelihood) and strength of association. Effect estimates were expressed as ORs. Potential confounding factors which do not vary over the short period of the study (eg, age, smoking and history of exacerbations) are controlled for by the case cross-over design. Other potential and time-variant confounders (eg, temperature and self-reported respiratory infections) were evaluated by adding variables into the model and observing changes in effect size.

## Results

We enrolled 246 patients with COPD into the study. Of these, 171 patients contributed data for at least one exacerbation and one control (non-exacerbation) period. We removed three patients because they had been hospitalised within a week prior to the call date suggesting that the 7-day window of the exposure questionnaire may not have captured the beginning of the exacerbation. We restricted our analyses to include the remaining 168 (68%) patients for whom we had adequate data. There were a total of 620 observations; 231 were exacerbation periods and 389 were control periods.

The mean age of the respondents was 70 years, 60% were female and almost all were white (table 1). Approximately two thirds of patients had severe or very severe COPD (GOLD grade III or IV). Ninety-six per cent of patients had a smoking history with an average of 52 pack years. Forty-three per cent of patients with COPD reported ever having a doctor diagnosis of asthma. Half reported working in a dusty job for at least 1 year and approximately 40% reported ever having been exposed to gas or chemical fumes at work. Ninety-nine per cent of patients reported ever having worked full time but only 14% reported working for at least 1 week during the study. Because of the low prevalence of currently employed participants, we did not investigate risk from recent occupational exposures.

**Table 1:** Characteristics of patients with COPD surveyed in the case cross-over study (n=168)

Age, mean years (SD)	70 (9.8)
Female, % (n)	60 (101)
Race:	
White	97% (163)
Black	1% (2)
Other	2% (3)
GOLD grade	
I (Mild)	2% (3)
II (Moderate)	23% (38)
III (Severe)	50% (82)
IV (Very Severe)	18% (30)
Normal FEV_1_/FVC with COPD symptoms	7% (12)
Inadequate spirometry	2% (3)
Reported doctor diagnosis of asthma	43% (73)
Smoking status	
Current	18% (30)
Ex	78% (131)
Never	4% (7)
Pack years (mean, SD)	52 (31)
FEV_1_ improvement post-bronchodilator >15%	20% (32)
Has ever worked full time (>30 hours per week)	99% (166)
Worked for at least 1 week of study period	14% (24)
Has worked for a year or more in dusty job	50% (83)
Severity of dusty job	
Mild	33% (27)
Moderate	40% (33)
Severe	28% (23)
Has ever been exposed to gas or chemical fumes in work	41% (68)
Severity of gas or chemical fume exposure	
Mild	37% (25)
Moderate	44% (30)
Severe	19% (13)

*Two observations were missing.

COPD, chronic obstructive pulmonary disease.

We first evaluated non-chemical triggers (weather, season, respiratory infection and exercise) (table 2). Self-reported respiratory infections or colds in the previous week were strongly associated with risk of exacerbation (OR=8.14, 95% CI: 4.67 to 14.2). Season was also associated with increased risk of exacerbation with fall having the strongest effect followed by spring and then winter. Strong effects were also observed for average outdoor temperatures in the previous week; the effect was strongly non-linear with higher risk for moderate (from 40°F to 50°F or from 4.4°C to 10°C) compared with cold temperatures (less than 40°F or 4.4°C) (OR=2.50, 95% CI: 1.51 to 4.12) and a protective effect for warm temperatures (greater than 50°F or 10°C) versus the same cold temperature range (OR=0.55, 95% CI: 0.36 to 0.84). The temperature and season variables performed fairly similarly with respect to overall model fit (similar values of Akaike Information Criteria-AIC). We chose the temperature variable for most models because it was more parsimonious and easier to interpret than season, which varies in meaning in different regions. These patterns are described in more detail in a previous paper.^34^

**Table 2:** Non-chemical triggers and risk of COPD exacerbation

Variable type	Variable description	OR	95% CI		−2log likelihood
Season	Season, *ref=summer*				417
	Summer	1.00	1.00	1.0	
	Spring	2.59	1.55	4.35	
	Winter	2.38	1.37	4.11	
	Fall	6.64	3.83	11.5	
Weather	Spent time in cold air/cold weather in past week^*^	1.16	0.78	1.70	468
	Spent time in hot air/hot weather in past week^*^	0.50	0.34	0.75	457
	Local temperature (categorical)^‡^				422
	Cold (<40°F)	1.0	1.0	1.0	
	Moderate (40–50°F)	2.50	1.51	4.12	
	Warm (>50°F)	0.55	0.36	0.84	
Acute illness	Respiratory infection/cold in past week^*^	8.14	4.67	14.2	396
Physical activity	Total hours spent outside of the home^*,§^	0.99	0.99	1.00	469
	Exercised in past week^*^	0.60	0.39	0.93	462

*Based on self-reports from exposure questionnaires.

†Based on 7-day averages using data from NOAA collected at Worcester Regional Airport.

‡Categorical representation of variable based on cut-offs identified in restricted cubic splines.

§Estimated as the sum of total ‘hours reported outdoors’, ‘hours reported in a vehicle’ and ‘hours reported indoors but not at home’.

COPD, chronic obstructive pulmonary disease.

Self-report of exercise (purposeful physical activity such as walking, bicycling or participating in an exercise class for physical fitness) in the previous week was negatively associated risk of exacerbation (OR=0.60, 95% CI: 0.39 to 0.93) and this factor was independent of temperature or season. We therefore adjusted for temperature, self-reported respiratory infections and exercise in models exploring potential environmental exposure risk factors.

**Figure 1:**
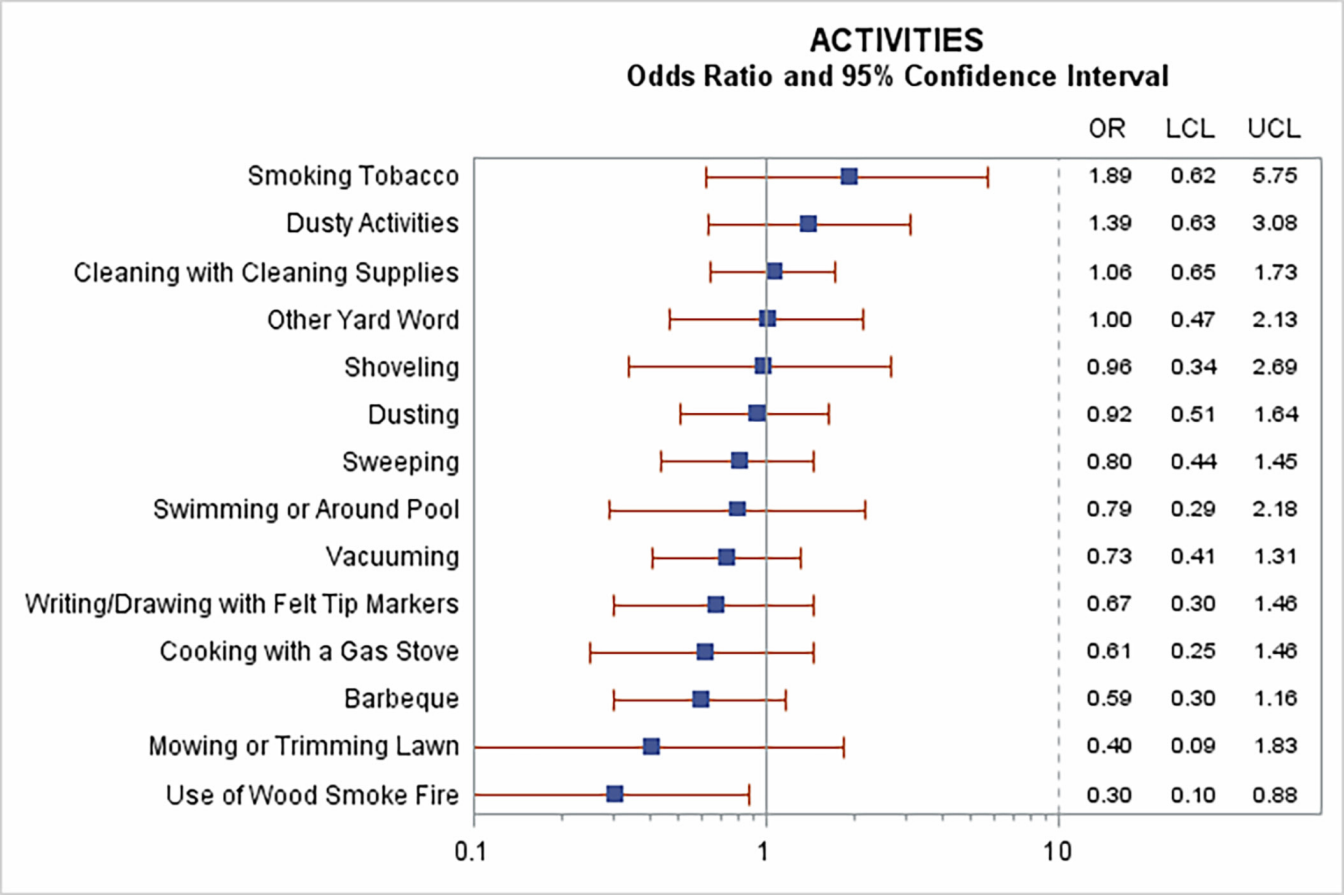
Associations between self-reported activities and risk of COPD exacerbation. Results of models including each activity alone and controlled for temperature, self-reported respiratory infection and exercise. COPD, chronic obstructive pulmonary disease; LCL, lower confidence limit; UCL, upper confidence limit)

The survey asked about a wide range of *physical activities* that might have resulted in irritant chemical exposures in the previous week (figure 1). Reports of smoking tobacco and ‘dusty activities’ were weakly associated with increased risk of exacerbation, although confidence intervals were wide and included the null. No other activities were associated with increased risk of exacerbation: one (use of a wood smoke fire) was associated with a *reduced* risk (OR=0.30, 95% CI: 0.10 to 0.88) when controlling for self-reported respiratory infection, exercise and temperature. Because wood fire use was predominantly reported in the winter (data not shown), we also fit this model with season instead of the temperature variable (as noted above, they generally performed similarly) and found that the apparent protective effect of wood smoke was weakened substantially with a CI that included the null. We therefore dropped this variable from further consideration.

**Figure 2:**
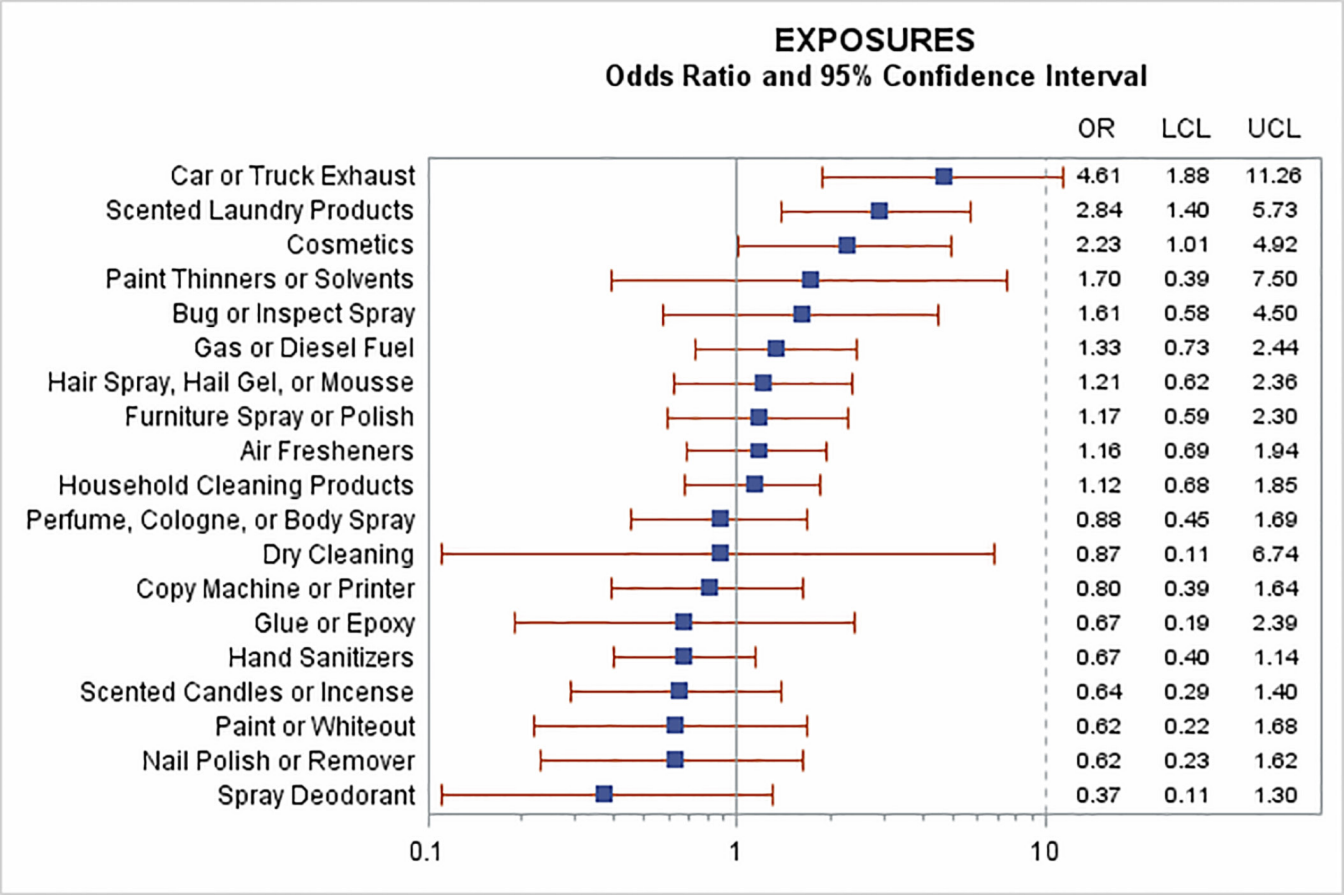
Associations between self-reported chemical exposures and risk of COPD exacerbation. Results of models including each activity alone and controlled for temperature, self-reported respiratory infection and exercise. COPD, chronic obstructive pulmonary disease.

The survey also gathered data on self-reported *exposures to products and chemicals* (figure 2). Among these, there was evidence of increased risk of COPD exacerbation from exposure to car or truck exhaust (OR=4.61, 95% CI: 1.88 to 11.26), use of scented laundry products (OR=2.84, 95% CI: 1.40 to 5.73) and cosmetics (OR=2.23, 95% CI: 1.01 to 4.92) (figure 2). The cosmetics effect was restricted to women, as no men reported this exposure.

Logistic regression models were constructed with these three exposures in addition to the three non-chemical factors (temperature, self-reported respiratory infection and exercise). Exposures to car/truck exhaust and scented laundry products did not appear to confound one another nor were they confounded by the three other variables (table 3). However, the effect of cosmetics became weaker with a 95% CI that included the null suggesting some possible confounding by one of the other factors. The cosmetics variable was therefore dropped from further consideration. In this model (table 3), self-reported respiratory infection had the strongest effect (OR=7.90, 95% CI 4.29 to 14.50). Car and truck exhaust and scented laundry products also showed strong positive effects (OR=4.36, 95% CI: 1.76 to 10.80; OR=2.69, 95% CI: 1.31 to 5.52, respectively). Temperature (moderate versus cold temperatures OR=1.95, 95% CI 1.09 to 3.49, and warm versus cold temperature OR=0.43, 95% CI: 0.26 to 0.70) also showed similar strong effects as in univariate models.

**Table 3:** Results of multivariate model of factors associated with risk of COPD exacerbation

Variable	OR	95% CI	
Car/truck exhaust	4.36	1.76	10.8
Scented laundry	2.69	1.31	5.52
Temperature			
warm versus cold	0.43	0.26	0.70
moderate versus cold	1.95	1.09	3.49
Self-reported respiratory infection	7.90	4.29	14.5
Exercise	0.59	0.35	1.00

AIC=351.

*2log likelihood=339.

COPD, chronic obstructive pulmonary disease.

In a previous paper,^34^ we investigated the hypothesis that urban air pollution measured at central monitors was associated with risk of exacerbation in this same population. We found that short-term exposures to sulfur dioxide (SO_2_) (OR=2.45, 95% CI: 1.75 to 3.45 for a one part per billion increase) was associated with an increase in COPD exacerbation risk. Surprisingly, there was a negative association with 7-day average PM_2.5_ (OR=0.91, 95% CI 0.85 to 0.98). We included these air pollution exposures in logistic regression models along with the factors described in the present investigation to evaluate potential confounding. The air pollution effects appeared to be independent of the associations reported in table 3, and neither the effects of self-reported chemical exposures nor the air pollution effects were importantly altered when all were included in the same model (see online supplementary appendix 1).

10.1136/bmjresp-2017-000179.supp1 Supplementary Appendix 1

## Discussion

Chronic disease management has been shown to improve symptoms, quality of life and other outcomes (emergency room visits and inpatient admissions) in patients with COPD.^7 8 35^ It is important that patients know and control triggers and symptoms of COPD exacerbations. To date, viral respiratory infections have been understood to be the primary trigger for exacerbations, and so prevention strategies have emphasised annual influenza and pneumonia vaccines and rapid treatment of exacerbations once they begin. Our findings support these recommendations. In particular, we found that self-reported respiratory infection was strongly associated with exacerbation.

We hypothesised that patients with COPD being closely followed as a part of a DMG would report irritant chemical exposures more frequently in the week leading up to a clinically confirmed exacerbation than in normal weeks. We therefore questioned participants about a wide range of potential triggers as an initial screening of this hypothesis. While most of the exposures and activities were not associated with exacerbation, several were: particularly car and truck exhaust and scented laundry products. We asked about exposure to fragrances in various forms and it is not clear, for example, why air fresheners or household cleaning products appeared not to be associated with exacerbation. Whether these associations are causal cannot be determined from this first investigation but we believe that these findings warrant follow-up.

There are several advantages of this study design. First, participants tend to be highly motivated and accustomed to interacting with a nurse as a part of their normal healthcare. Second, we were able to detect and clinically confirm exacerbations that may be less severe than those that result in hospital admissions. Indeed, a major goal of the DMG is to keep patients out of the hospital. Also, there is probably often a lag period of days between any triggering exposure and the ultimate hospital admission. Our study probably shortened the delay between exposure and identification of the exacerbation by taking advantage of the normal routine of the DMG, in which patients were instructed to call the nurse at the first signs of an exacerbation.

Another strength of this study was that the case cross-over design essentially uses each participant ‘as their own control’ by comparing case and control time periods within the same individual.^25^ A consequence is that any ‘fixed’ characteristic of the participants (eg, gender, age, smoking history and exacerbation history) is controlled for by design. This allows the study considerable power to detect short-term variable exposures like air pollution that might be associated with the onset of an exacerbation.^36–39^ While other studies demonstrate this association in emergency room and hospitalisation data, our evidence may be stronger because of the greater sensitivity of the case cross-over that includes not only those exacerbations that progress to emergency room or inpatient care.

There are also limitations of the study. We necessarily relied on patient recall for exposure information and so recall bias is a possibility when comparing exacerbation periods to control periods. However, the finding that only a few exposures were more frequently reported in exacerbation weeks than normal weeks provides evidence against the possibility that participants were non-discriminately over-reporting all exposures because of their beliefs about the causes of their illness. As is common in a pilot investigation, we asked about a wide range of exposures, and so many statistical comparisons were made; thus, caution is appropriate when interpreting the precision of effect estimates. Although approximately 43% of patients self-reported a history of doctor diagnosed asthma, we did not have enough statistical power to conduct stratified analyses in this cohort. This is unfortunate because one might speculate that patients with COPD with comorbid asthma might respond differently than patients with COPD without asthma to certain exposures such as sensitisers or irritants.

Although we believe that the design may have reduced the lag between exposure and exacerbation in comparison to hospital admissions studies, we nevertheless found evidence that suggested that participants may be aware of a worsening of their symptoms for some days before they start medications and call the nurse for clinical confirmation. If this is true, then they may also, even unconsciously, start avoiding certain triggering exposures. We found evidence suggesting that this may have occurred. For example, participants were less likely to report exercise in the week preceding an exacerbation compared with a normal week (table 2). These reductions may have occurred because the participant was starting to have symptoms and was reducing activities as a result. Models controlling for these variables did not change the findings; however, the bias may still have occurred.

Before the study began, we conducted focus groups of patients with COPD and learnt that many of them held well-formed opinions about chemical exposures and activities which they seek to avoid, managing their disease and reducing risk of exacerbation. Some clinicians also support the idea that exacerbations can be prevented through avoidance of certain common exposures, for example, cigarette smoke, exhaust fumes, dust, strong odours and household cleaning products,^40^ despite the fact that little or no direct evidence exists to support these recommendations.

The strongest evidence in this study suggests that self-reported exposures to car and truck exhaust and scented laundry products were associated with an increased risk of COPD exacerbation. These findings were not confounded by any other known risk factors including having a respiratory infection or cold, temperature or outdoor air pollutants.

This case cross-over study found that the risk of COPD exacerbation was increased by certain environmental chemical exposures. These findings should be followed-up with investigations using quantitative exposure assessments to strengthen evidence that could help patients with COPD manage their disease and improve their quality of life.
