# Supplementary material for: Environmental triggers of COPD symptoms: a case cross-over study
Source: BMJ Open Respir Res. 2017 Jul 3;4(1):e000179. doi: 10.1136/bmjresp-2017-000179 (PMC5647479; doi:10.1136/bmjresp-2017-000179)
Supplement: Supplementary Appendix 1 [file bmjresp-2017-000179supp001.pdf]

APPENDIX

**Appendix Table 1. Results of multivariate model of factors associated with risk of COPD exacerbation including air pollutants**			
**Variable**	**OR**	**95% CI**	
Car/Truck Exhaust	5.81	2.15	15.7
Scented Laundry	2.93	1.36	6.29
Temperature based on spline			
Warm versus Cold	0.75	0.44	1.27
Moderate versus Cold	2.22	1.20	4.11
Self Reported Resp. Infection	7.57	3.95	14.5
Exercise	0.56	0.32	0.97
SO_2; 7 day local average	2.66	1.85	3.81
$PM_{2.5}$; 7 day local average	0.92	0.85	0.99

AIC = 321

-2log likelihood = 305
